# Associations between socioeconomic status and blood cadmium levels in Korea

**DOI:** 10.4178/epih.e2019018

**Published:** 2019-05-15

**Authors:** Yongho Jee, Sung-il Cho

**Affiliations:** Department of Public Health Science, Graduate School of Public Health, Seoul National University, Seoul, Korea

**Keywords:** Cadmium, Smoking, Socioeconomic status, Korea National Health and Nutrition Examination Survey

## Abstract

**OBJECTIVES:**

Although smoking is associated with both low socioeconomic status and blood cadmium (Cd) levels, the association between socioeconomic status and Cd levels remains unclear. Therefore, our study aimed to examine this association and to clarify whether smoking is a confounding or mediating variable in this relationship.

**METHODS:**

Data (n=7,734) were drawn from the Korea National Health and Nutrition Examination Survey (KNHANES, 2008- 2011), including years that contained data on blood Cd and urinary cotinine levels. We investigated the associations of income, education, and occupation with blood Cd levels. Smoking was investigated by categorizing participants by smoking status (never, former, and current) and pack-years into quartiles. The weekly frequency of rice and barley intake was analyzed to gain insights into participants’ dietary patterns. Additionally, urinary cotinine levels were used to ensure the validity of the smoking variables.

**RESULTS:**

Participants earning a low income and with less formal education had higher blood Cd levels. After controlling for smoking, the association between income and Cd levels substantially weakened. Further controlling for education, the association between income and Cd levels disappeared. However, there was a strong negative association between education and Cd levels, even after controlling for smoking history, pack-years, and urinary cotinine levels.

**CONCLUSIONS:**

In cross-sectional data from the KNHANES, blood Cd levels were significantly higher among those with a low income and less formal education. Smoking history contributed to, but did not fully explain, these associations.

## INTRODUCTION

Cadmium (Cd) is well known as a heavy metal used in our daily life, occupational and environmental exposure to which is harmful to the human body. Cd is toxic even at low exposure levels, and has acute and chronic effects on health and the environment [1]. Since Cd is not biodegradable, it remains in circulation once released into the environment [2-4]. The routes through which humans are exposed to Cd are commonly categorized as occupational exposure and environmental exposure (non-occupational exposure). The occupational route involves exposure to Cd by the mining, production, and consumption of non-ferrous metal or plating, welding, the utilization of storage batteries and agricultural chemicals, and pesticide production [5]. Environmental exposure to Cd most often occurs through food intake such as meat, shellfish, vegetables, and any form of food whose packaging contains Cd; moreover, exposure to cigarette smoking and dust from polluted soil are routes of chronic exposure [6,7]. In Korea, smoking prevalence was extremely high in the 2000s. According to a report by Jee et al. [7], the prevalence of smoking among males in Korea in 2000 was 68%. Furthermore, rice consumption is considered to be a major factor associated with high Cd levels in Asian countries, including Korea [8].

The environmental route usually involves lower levels of exposure, and therefore a lower risk of resulting disease, than occupational exposure. However, some health effects have been reported to be associated with low-level exposure, such as blood pressure elevation and kidney function degradation. Furthermore, since environmental exposure to Cd involves numerous aspects of daily activities, is globally recognized as a public health hazard, and may disproportionately affect vulnerable groups such as children, pregnant females, and the elderly, the potential effects of Cd exposure should be a concern from a public health perspective.

However, few studies have examined the associations of socioeconomic variables such as income level, education level, and occupation with Cd levels, together with an analysis of the possible confounding effect of smoking.

## MATERIALS AND METHODS

### Subjects

The data used in this study were drawn from the fourth and fifth waves of the Korea National Health and Nutrition Examination Survey (KNHANES IV and V, 2007-2012) conducted by the Korean Centers for Disease Control and Prevention (KCDC) and the Ministry of Health and Welfare, a nationally representative sample recruited using a multi-stage clustered probability design. The data included respondents from 20 years to 87 years of age. Children and adolescents were excluded since only adults received testing for heavy metal levels. The data were obtained from 2 independent cross-sectional waves of the KNHANES (2007-2009, 2010-2012), and were limited to years that contained data on blood Cd and urinary cotinine levels (2008, 2009, 2010, 2011). The sample size of the participants with data blood Cd and urinary cotinine levels was 7,734.

### Study variables

Education level, income, and occupation were included as socioeconomic factors in this study, following previous studies by Tyrrell et al. [9] and McKelvey et al. [10]. Participants were classified by age into 5 groups (20-29, 30-39, 40-49, 50-59, and ≥60 years). Education level was classified into 4 groups by each participant’s final level of schooling (less than elementary school graduate, middle school graduate, high school graduate, and college graduate). Occupation was originally categorized into 7 groups (managers, clerical workers, service workers, agricultural, fishery workers, craft workers, elementary workers, and unemployed) based on the Korean Standard Classification of Occupations of the Korea National Statistical Office. However, for the present study, occupations were reclassified into 4 categories (professional and managers; non-manual, skilled, and semi-skilled workers; unskilled workers; and unemployed), following the classification used in the Whitehall study [11].

### Blood cadmium levels

The average blood Cd level was 1.24 μg/L (standard deviation [SD], 1.24; range, 0.02-8.34) for male and 1.57 μg/L (SD, 0.62; range, 0.02-6.03) for female. Cd levels were not normally distributed; instead, the data were left-skewed. Therefore, log-transformed values or geometric mean values were used for the analysis in this study.

### Statistical analysis

A complex sampling design was used for the KNHANES data, including stratification, clusters, and weights to represent the entire Korean population. The demographic characteristics and the prevalence are presented as weighted percentages to describe nationally representative data, as calculated using PROC SURVEYMEANS and PROC SURVEYREG. Initially, descriptive statistics were computed for all study variables, including frequencies and percentages for categorical variables, and means and SDs for continuous variables.

Next, we investigated the association of Cd levels with income by comparing the Cd levels between the highest income quartile (Q4) and the lowest income quartile (Q1). The association between Cd levels and education was investigated by comparing blood Cd levels between college graduates and those who had completed an elementary-school education or less. Occupational associations were investigated by using the occupation classification variable of the KNHANES to compare Cd levels between professionals and managers and the unemployed. Associations between Cd levels and smoking were investigated by categorizing current smoking status, smoking amount, smoking duration, and pack-years into quartile variables. Additionally, urinary cotinine levels were used to ensure the validity of the smoking variables. SAS version 9.4 (SAS Institute Inc., Cary, NC, USA) was used for preliminary analyses, and Mplus version 7.4 (https://mplus.software.informer.com/7.4/) was used for the path analyses [12].

### Ethics statement

Data from the KNHANES survey are made publicly available through the KNHANES website (http://knhanes.cdc.go.kr). Thus, ethical approval was not required for this study. All participants provided written informed consent.

## RESULTS

### General characteristics of the study participants

The general characteristics of the KNHANES study participants are shown in Table 1. The differences in the age and sex distribution by year were negligible. However, the distribution of college graduates increased from 27.6% in 2008 to 35.8% in 2011.

The distribution of the general characteristics of participants stratified by sex is presented in Table 2. The sample size of female (n=3,913, 51.0%) was slightly larger than that of male (n=3,821, 49.0%). The average body mass index was higher in male than in female. The average cotinine level and number of current smokers among male was approximately 7 times higher than among female. The proportion of participants with an elementary education or less was higher in female (25.5%) than in male (13.5%). Furthermore, approximately twice as many of the unemployed participants were female (50.2%) than male (22.5%).

### Association between income and blood cadmium levels

Table 3 demonstrates trends in the association between income and blood Cd levels by sequentially controlling for other variables, including sex, smoking status, education level, and occupation. A basic model comparing blood Cd levels by income level without sex stratification is shown in model 1. The log-transformed blood Cd level in the lowest income group (Q1) was 0.108 μg/L higher than in the highest income group, which was the reference group (model 1). When sex was further controlled, the association remained stable. However, after additionally controlling for smoking status, the association was somewhat attenuated, but remained significant. A sharp fluctuation occurred after controlling for education level, at which point the association between income and blood Cd levels was totally eliminated. Therefore, the association between income and blood Cd levels was confounded by smoking status and education level.

### Path analysis of the associations of age, income, education, and smoking pack-years with blood cadmium levels

**Figure 1.**
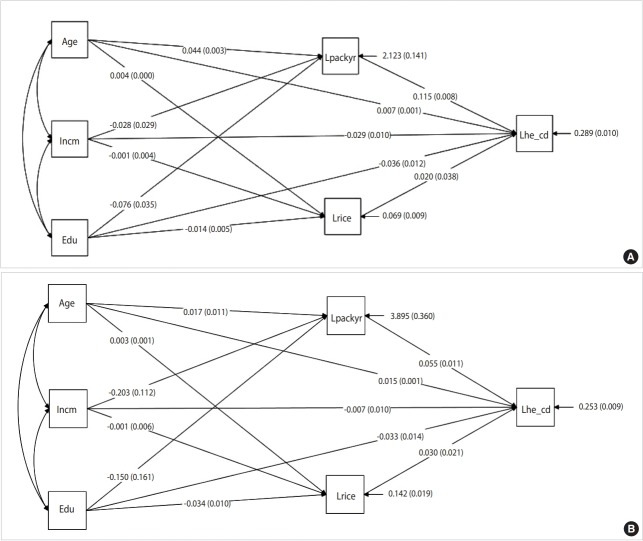
Path analysis of the overall associations of blood cadmium levels with smoking pack-years and social determinants including income level, education level, and occupation classification in (A) male and (B) female. Values are presented as β (p-value). Lpackyr, logtransformed pack year; Incm, income; Lhe_cd, log-transformed cadmium; Edu, education; Lrice, log-transformed rice.

Figure 1A presents the results of a path analysis of the overall association of blood Cd levels with smoking pack-years and social determinants including income level, education level, and occupation classification in male. Smoking pack-years, income level, education level, and age all demonstrated significant associations with blood Cd levels. The association between income and smoking pack-years was -0.028, which supported our hypothesis. The direct relevance of education level for blood Cd levels was -0.036, while its indirect relevance to blood Cd levels by going through smoking pack-years was -0.009 (=-0.076×0.115).

Figure 1B presents the results of a path analysis of the overall association of blood Cd levels with smoking pack-years and social determinants including income level, education level, and occupation classification in female. Unlike the results for male, smoking pack-years, education level, and age were significantly associated with blood Cd levels. However, income did not show a significant association.

## DISCUSSION

In this analysis of cross-sectional data from the KNHANES, we aimed to explain the association between blood Cd levels and income using a modeling method in which factors related to Cd levels, such as sex, smoking status, education, and occupation, were added step by step. Blood Cd levels were significantly higher among those with low income and less formal education. However, these associations were substantially attenuated after controlling for smoking status, although they still remained present. The results of this study suggest that blood Cd levels may also be a biomarker reflecting socioeconomic disparities. Moreover, interventions targeting Cd levels in individuals in the low-income and low-education groups who are highly exposed to Cd are needed. The results of our analysis correspond with existing results regarding health inequities and heavy metal exposure [9,10]. In this study, an association between income and blood Cd levels was found, but the magnitude of this association decreased from 0.126 to 0.050 in male and from 0.076 to 0.057 in female after controlling for smoking status. The sharp decrease in the magnitude of the association among male after controlling for smoking status seems to reflect the close association between smoking status and blood Cd levels. Therefore, the association between income and blood Cd levels can be interpreted as the result of confounding by smoking status. However, the relatively small decrease in the magnitude of the association among female after controlling for smoking could be interpreted in light of previous studies reporting the high prevalence in Cd level among female [13]. However, it seems more likely that the results in our study are due to the low reliability of self-reported smoking among female, as a previous study reported unrealistically low rates of smoking prevalence in Asian female due to under-reporting [14]. Although we replaced the self-reported smoking variable among female with urinary cotinine levels, the results were similar after controlling for urinary cotinine levels, which can be interpreted as indicating that confounding effect of urinary cotinine levels was lower among female than among male.

Therefore, smoking made a significant contribution to the association between income and blood Cd levels in male, but not in female. In order to analyze the remaining association between income and blood Cd levels, education level and occupation classification were additionally controlled. Doing so eliminated the association between income and blood Cd levels. Integrating these findings, the association between income and blood Cd levels appears to result from confounding by smoking status and education level.

Previous studies have investigated associations between smoking status and Cd exposure. According to a recent meta-analysis reported by Hecht et al. [15], the correlation coefficient between cigarette smoking and Cd levels was 0.540. The associations found between cigarette smoking and Cd levels were 0.546 in this study (0.663 in male and 0.316 in female). Moreover, the present study demonstrated this association not only with cigarette smoking as a self-reported categorical variable, but also with pack-years and urinary cotinine levels, making our results more sophisticated than those of previous studies.

Female tended to have higher blood Cd levels in this study, and several former studies have reported that the health effects of toxic metals differ in prevalence or manifest differently in male and female [13]. Vahter et al. [13] reported that Cd-related health effects were more common among female than male, and hypothesized that this may have been due to differences in sensitivity to toxic effects; however, it remains unclear whether that is the case, as it is possible that their findings merely reflected Itai-itai disease, which involves a combination of kidney damage, osteomalacia, and osteoporosis, is the most advanced form of Cd-induced disease, and it has been reported to be caused by consumption of rice that is heavily contaminated by Cd emitted from mines [16,17]. A previous study also found that Itai-itai disease more strongly affected elderly, multiparous female; however, the degree to which those findings could be interpreted as showing a causal relationship relevant for risk assessment is limited [18].

Several studies have reported that Cd exerts estrogenic effects [14-19]. Based on those results, Cd was found to mimic estrogens in breast cancer cells as a result of its ability to form a high-affinity complex with the hormone-binding domain of the estrogen receptor [20].

One of the major strengths of this study is its use of the KNHANES, a representative data source. The KNHANES is a cross-sectional, nationally-representative survey of the general population in Korea, annually conducted by the KCDC as part of the Ministry of Health and Welfare. Although the design was cross-sectional, the risk of an inverse association was relatively low, because socioeconomic determinants are formed throughout a long-term period, and blood Cd accumulates for a long period in the human body. Although smoking is associated with both low socioeconomic status and blood Cd levels, the association between socioeconomic status and Cd levels remains unclear. In cross-sectional data from the KNHANES, blood Cd levels were significantly higher among those with a low income and less formal education. Smoking history contributed to these associations, but did not explain them entirely.

## Figures and Tables

**Table 1.** General characteristics of the study subjects stratified by year, KNHANES

Characteristics	2008	2009	2010	2011
Total participants (n)	9,744	10,533	8,958	8,518
Study participants (n)	1,936	1,964	1,916	1,918
Sex, male (%)	49.9	50.0	49.3	49.5
Age, mean±SD (yr)	45.5±15.5	45.5±15.5	45.3±14.5	45.1±14.5
Education				
Less than primary	455 (22.7)	419 (21.2)	359 (18.5)	310 (15.9)
Middle school	242 (12.1)	225 (11.4)	203 (10.5)	209 (10.7)
High school	751 (37.5)	748 (37.8)	694 (35.8)	736 (37.7)
College or more	533 (27.6)	587 (29.7)	683 (35.2)	700 (35.8)
Occupation				
Managers	229 (11.5)	261 (13.2)	283 (14.5)	292 (14.9)
Clerical workers	142 (7.1)	182 (9.2)	211 (10.9)	181 (9.3)
Service workers	250 (12.6)	272 (13.8)	274 (14.2)	265 (13.6)
Agricultural, fishery	156 (7.8)	114 (5.8)	155 (8.0)	114 (5.8)
Craft workers	213 (10.7)	229 (11.6)	217 (11.3)	243 (12.4)
Elementary	199 (10.0)	179 (9.1)	137 (7.1)	157 (8.0)
Unemployed	801 (40.3)	738 (37.4)	651 (33.8)	702 (35.9)
Income (quantile)				
Bottom (Q1)	497 (25.5)	512 (25.9)	506 (26.1)	466 (23.7)
Second (Q2)	478 (24.5)	497 (25.2)	494 (25.5)	509 (23.7)
Middle (Q3)	491 (25.2)	483 (24.5)	469 (21.2)	491 (25.0)
Fourth (Q4)	485 (24.9)	483 (24.5)	470 (24.2)	502 (25.5)

Values are presented as number (%). KNHANES, Korea National Health and Nutrition Examination Survey (a nationally representative sample recruited using a multi-stage clustered probability design); SD, standard deviation.

**Table 2.** Characteristics of study participants with data on blood Cd levels and cotinine levels, KNHANES (n=7,734)

Variables	Total	Weighted	Blood Cd (μg/L)^1^ GM (95% CI)	p-value
Age (yr)				
20-29	1,493 (19.3)	3,497,948 (27.8)	1.00 (reference)	
30-39	1,568 (20.3)	2,646,615 (21.0)	0.87 (0.84, 0.91)	<0.001
40-49	1,571 (20.3)	2,778,299 (22.1)	1.08 (1.05, 1.11)	<0.001
50-59	1,549 (20.0)	2,235,992 (17.7)	1.22 (1.18, 1.25)	<0.001
≥60	1,553 (20.1)	1,438,462 (11.4)	1.24 (1.20, 1.27)	<0.001
Sex				
Male	3,821 (49.4)	7,121,327 (56.5)	1.00 (reference)	
Female	3,913 (50.6)	5,475,988 (43.5)	1.01 (0.98, 1.03)	<0.001
Education				
Elementary or less	1,512 (19.5)	1,735,973 (13.8)	1.00 (reference)	
Middle school	860 (11.1)	1,247,189 (9.9)	1.18 (1.14, 1.24)	<0.001
High school	2,877 (37.2)	5,258,149 (41.7)	0.88 (0.85, 0.90)	<0.001
University or more	2,485 (32.1)	4,356,004 (34.6)	0.77 (0.75, 0.79)	<0.001
Household income				
Q4 (high)	1,950 (25.2)	3,271,804 (26.0)	1.00 (reference)	
Q3	1,955 (25.3)	3,182,180 (25.3)	0.89 (0.86, 0.92)	0.095
Q2	1,905 (24.6)	3,102,698 (24.6)	0.94 (0.91, 0.97)	<0.001
Q1 (low)	1,924 (24.9)	3,040,633 (24.1)	0.97 (0.94, 1.01)	<0.001
Occupation (n=7,707)				
Professionals and managers	1,055 (13.7)	1,915,934 (15.3)	1.00 (reference)	
Non-manual, skilled workers	3,179 (41.2)	5,408,467 (43.1)	0.94 (0.92, 0.98)	<0.001
Unskilled workers	660 (8.6)	1,037,897 (8.3)	1.09 (1.04, 1.15)	<0.001
Unemployed	2,813 (36.5)	4,176,645 (33.3)	0.90 (0.87, 0.93)	<0.001
Smoking status				
Never	4,166 (53.9)	6,137,777 (48.7)	1.00 (reference)	
Former	1,596 (20.6)	2,603,822 (20.7)	0.77 (0.74, 0.80)	<0.001
Current	1,972 (25.5)	3,855,715 (30.6)	1.12 (1.09, 1.16)	<0.001

Values are presented as number (%). Cd, cadmium; KNHANES, Korea National Health and Nutrition Examination Survey; GM, geometric mean; CI, confidence interval.

1 Age-adjusted GM with CI.

**Table 3.** Associations between income level and log-transformed blood cadmium levels (n=7,707)^1^

Variables		Model 1	Model 2	Model 3	Model 4	Model 5
Income level	Q4 (high)	Reference				
	Q3	0.037 (0.112)	0.032 (0.163)	0.014 (0.511)	0.001 (0.969)	0.001 (0.946)
	Q2	0.081 (<0.001)	0.078 (<0.001)	0.046 (0.033)	0.022 (0.317)	0.022 (0.309)
	Q1 (low)	0.108 (<0.001)	0.102 (<0.001)	0.046 (0.042)	0.007 (0.776)	0.009 (0.682)
Sex	Male		Reference	Reference	Reference	Reference
	Female		0.146 (<0.001)	0.384 (<0.001)	0.371 (<0.001)	0.376 (<0.001)
Smoking status	Never smoker			Reference	Reference	Reference
	Ex-smoker			0.056 (0.018)	0.057 (0.015)	0.057 (0.016)
	Current smoker			0.546 (<0.001)	0.535 (<0.001)	0.533 (<0.001)
Education	University graduate or more	4			Reference	Reference
	High school graduate	3			0.087 (<0.001)	0.085 (<0.001)
	Middle school graduate	2			0.144 (<0.001)	0.140 (<0.001)
	Elementary graduate or less	1			0.180 (<0.001)	0.176 (<0.001)
Occupation	Professionals and managers	1				Reference
	Non-manual, skilled workers	2				0.020 (0.380)
	Unskilled workers	3				- 0.008 (0.801)
	Unemployed	4				-0.009 (0.685)
R^2^		0.205	0.219	0.336	0.343	0.344

Values are presented as β (p-value).

1 Adjusted for age (20-29, 30-39, 40-49, 50-59, and ≥60 years), income level, sex, smoking status, education, and occupation.
